# Lésions bulleuses et purpuriques unilatérales: pathomimie cutanée

**DOI:** 10.11604/pamj.2015.20.301.5048

**Published:** 2015-03-30

**Authors:** Mouna Zinoun, Soumia Chiheb, Farida Marnissi, Nadia Kadiri, Hakima Benchikhi

**Affiliations:** 1 Service de Dermatologie-Vénéréologie, Centre Hospitalier Universitaire Ibn Rochd, Casablanca, Maroc; 2 Service d'Anatomopathologie, Centre Hospitalier Universitaire Ibn Rochd, Casablanca, Maroc; 3 Service de Psychiatrie, Centre Hospitalier Universitaire Ibn Rochd, Casablanca, Maroc

**Keywords:** Pathomimie cutanée, troubles factices, lésions bulleuses, dépression, thérapie cognitivo-comportementale, skin pathomimicry, factitious disorders, bullous lesions, depression, cognitive behavioral therapy

## Abstract

La pathomimie cutanée est une forme particulière de troubles factices relativement rare, et constitue l'un des problèmes les plus complexes pour le dermatologue. Nous rapportons un cas de pathomimie révélée par des lésions cutanées unilatérales, mimant une brûlure. Une jeune femme de 27 ans, était suivie depuis 4 ans pour une dépression. Elle a présenté 15j avant sa 1^ère^ hospitalisation un placard inflammatoire du sein gauche compliqué de lésions bulleuses et d’érosions superficielles. La biopsie cutanée avait montré une dermite non spécifique. Une cicatrisation rapide sous traitement local a été notée. Elle a présenté 10 jours plus tard de nouvelles lésions similaires étagées au membre inférieur gauche, évoluant vers le décollement bulleux spontané. La biopsie cutanée avait montré un décollement bulleux jonctionnel et des foyers de nécrose ischémique. L'IFD était négative. Devant les données anamnestiques, cliniques, la négativité du bilan paraclinique, et la guérison des lésions sous pansements occlusifs seuls, le diagnostic de pathomimie a été évoqué et retenu. La patiente a été adressée en psychiatrie où une thérapie cognitivo-comportementale a été préconisée. Notre observation correspond à un tableau de pathomimie de présentation clinique particulière par sa localisation unilatérale et son caractère bulleux. Chez notre patiente qui est droitière, la localisation unilatérale gauche sur des zones accessibles, l'absence de lésions spécifiques à l'examen histologique, la cicatrisation rapide des lésions sous traitement local occlusif seul et leur récurrence malgré des soins adaptés étaient en faveur d'une pathologie factice. Néanmoins, la localisation au niveau des seins peut être très déroutante. Le caractère bulleux des lésions dans le cadre d'une pathomimie a été rarement rapporté. Dans notre cas, la pathomimie s'associe à des troubles anxieux et dépressifs très importants. Leur prise en charge demande un investissement pluridisciplinaire le plus précoce possible. La prise en charge des pathomimies est complexe. Le traitement médical associé à une prise en charge psychologique de type thérapie cognitivo-comportementale, qui est une première expérience, peut aider cette patiente à contrôler son comportement et éviter les récidives qui sont fréquentes dans ce type de pathologie.

## Introduction

La pathomimie cutanée est une forme particulière de troubles factices relativement rare, provoquée par le patient lui-même sur son revêtement cutanéo-muqueux ou ses phanères, pour combler un besoin psychologique, dont il n'a pas conscience [1–3]. C'est l'un des problèmes les plus complexes pour le dermatologue car il doit accompagner progressivement le patient sans jamais le forcer à trahir son secret. L'anxiété, la dépression, les troubles de la personnalité accompagnent fréquemment la pathomimie et peuvent être un moyen pour justifier le recours au psychiatre [3]. Nous rapportons un cas de pathomimie révélée par des lésions cutanées unilatérales particulières.

## Patient et observation

**Figure 1:**
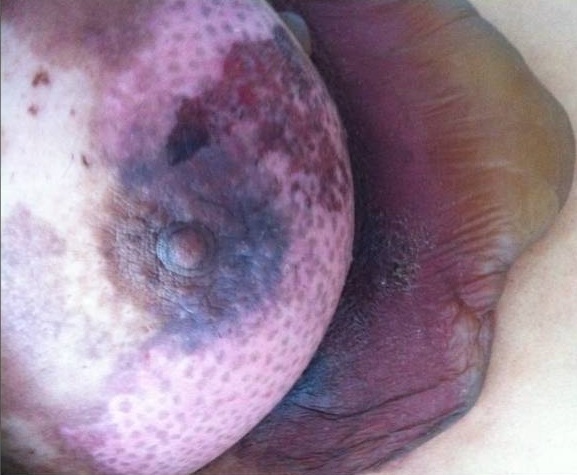
Lésions bulleuses et érosives débutantes du sein

Une jeune femme de 27 ans, célibataire, suivie depuis 4 ans pour une dépression survenue suite au décès de son père d'un cancer de la prostate; mise sous antidépresseurs et neuroleptiques sans nette amélioration. Elle a présenté 15 jours avant sa 1^ère^ hospitalisation un placard inflammatoire douloureux du sein gauche compliqué de lésions bulleuses et suivies d’érosions superficielles du quadrant inféro-externe et interne du sein gauche (Figure 1), associées à des plaques pigmentées post-inflammatoires avec un écoulement séreux multipore du mamelon gauche. Il n'y avait pas de nodule palpable ni d'adénopathies, le tout évoluant dans un contexte d'apyrexie et de conservation de l’état général.

**Figure 2:**
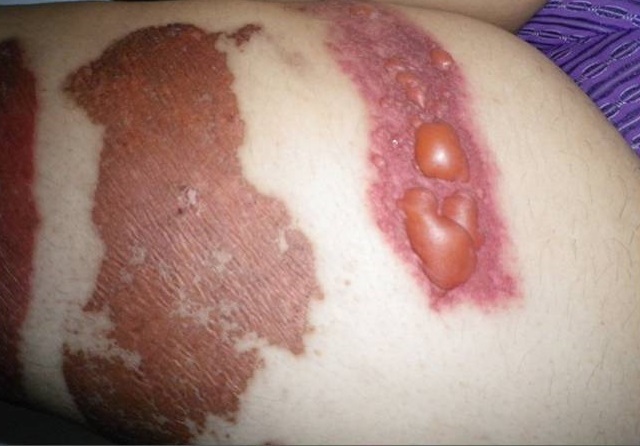
Lésions bulleuses sur un placard érythémateux violacé et purpurique

**Figure 3:**
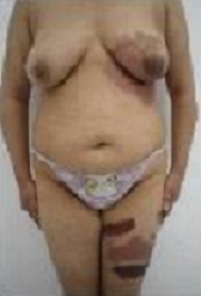
Remarquer l'atteinte unilatérale gauche des lésions

**Figure 4:**
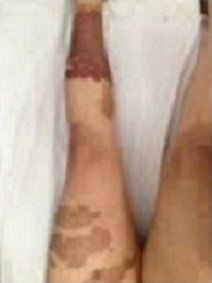
Lésions érythémato-purpuriques circonférentielles de la jambe gauche avec aspect cicatricielle des anciennes lésions

**Figure 5:**
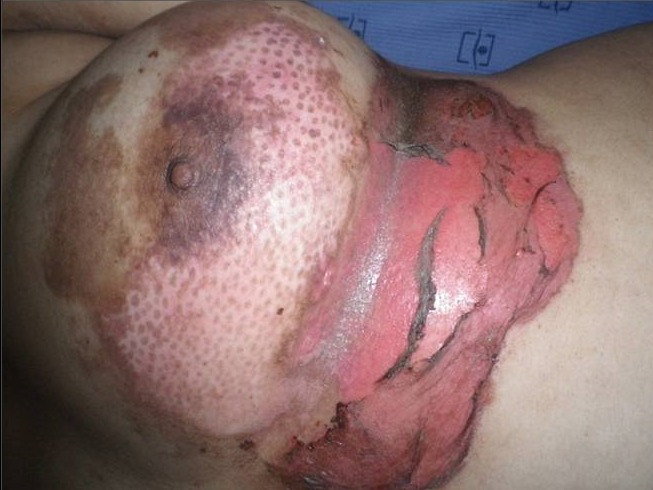
Érosion post-bulleuse

**Figure 6:**
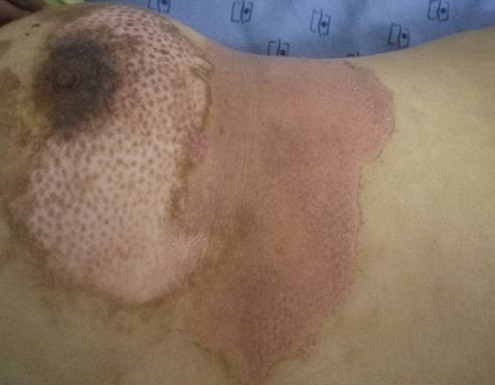
Lésions dyschromiques cicatricielles

**Figure 7:**
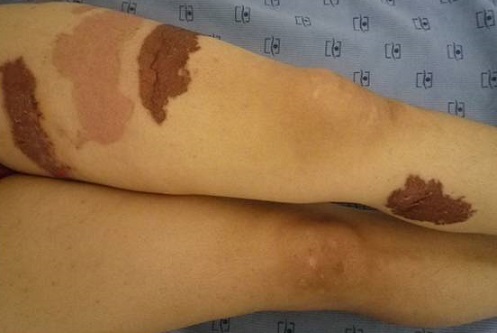
Coexistence de lésions d’âge différent

Les diagnostics évoqués étaient: un érysipèle bulleux, une mastite carcinomateuse, un érythème pigmenté fixe bulleux. Le bilan biologique n'avait pas retrouvé de syndrome inflammatoire, ni d'hyperleucocytose. Les prélèvements bactériologiques et mycologiques étaient stériles. La biopsie cutanée avait montré une dermite non spécifique. L’échographie mammaire était sans anomalies. La prolactinémie était normale. L’évolution a été marquée par la cicatrisation complète et rapide sous traitement local et antibiothérapie orale. La patiente a été réhospitalisée 10 jours plus tard pour l'apparition de nouvelles lésions étagées au membre inférieur gauche (cuisse et mollet), à type de plaques linéaires et à limites géométriques érythémato-violacées et purpuriques d'environ 15 cm de diamètre, évoluant vers le décollement bulleux spontané (Figure 2, Figure 3, Figure 4), associées à une érosion post-bulleuse du sein gauche (Figure 5) et à des lésions dyschromiques cicatricielles (Figure 6, Figure 7). Des cicatrices linéaires hypochromiques bilatérales des avant-bras et des poignets ont été constatées, probablement en rapport avec une automutilation. Le reste de l'examen était sans particularités. Le bilan biologique n'avait pas retrouvé de syndrome inflammatoire. Les sérologies virales (B, C, VIH) étaient négatives. Le cytodiagnostic de Tzanck n'avait pas objectivé de cellules acantholytiques ou ballonisantes. La biopsie cutanée avait montré un décollement bulleux jonctionnel, des foyers de nécrose ischémique siègeant au niveau du toit avec présence de cellules kératinocytaires apoptotiques. Cet aspect morphologique avec la négativité de l'IFD faisait discuter un érythème pigmenté fixe ou une dermite de contact. L’évolution était marquée par une cicatrisation rapide de toutes les lésions sous traitement local uniquement et un antihistaminique de 1^ère^ génération, sans apparition d'autres lésions au cours de son hospitalisation. Par ailleurs, l'interrogatoire avait révélé un milieu familial conflictuel avec des troubles du sommeil, une asthénie importante, une anxiété et une grande fragilité émotionnelle à l’évocation du décès de son père. Devant les données cliniques, la négativité du bilan paraclinique, les données de l'interrogatoire, la guérison des lésions sous pansements occlusifs seuls, le diagnostic de pathomimie a été évoqué et retenu. Devant le contexte de dépression, la patiente a été adressée en psychiatrie où ce diagnostic a été confirmé et la patiente a été mise sous antidépresseurs. Le caractère récurrent de cette pathologie nous a fait opté pour une thérapie cognitivo-comportementale pour éviter les récidives. Cependant, notre patiente a été perdue de vue et n'a pas continué le suivi psychiatrique.

## Discussion

Il s'agit d'un tableau de pathomimie de présentation clinique particulière par sa localisation unilatérale et le caractère bulleux des lésions, qui a posé initialement un problème diagnostique et thérapeutique. Dans notre cas, le diagnostic de pathomimie a été retenu devant un faisceau d'arguments cliniques, et la négativité des données paracliniques avec l'absence de lésions spécifiques histologiques éliminant une dermatose connue, dans un contexte social conflictuel particulier. Les pathomimies se définissent comme une maladie autoprovoquée dans un état de conscience claire par le patient lui-même, au niveau de son revêtement cutanéo-muqueux ou de ses phanères [1]. Elle constitue l'un des problèmes diagnostiques et thérapeutiques les plus complexes en dermatologie. Elle reste relativement rare et représente 2% de la pathologie dermatologique clinique [4]. Les pathomimes sont en général des femmes, avec un sexe ratio femme/homme de 3/1 à 20/1 [4–6]. Elle est plus fréquente chez les adolescents et les adultes jeunes, surtout célibataires. Tel était le cas chez notre patiente. Les aspects cliniques cutanés sont très variés à type de plaies, brûlures, abcès, granulomes à corps étrangers, érythèmes, ecchymoses, saignements, eczémas, dermatoses autoaggravées, sans que cette liste soit limitative. Les excoriations, les ulcérations, les lésions purpuriques et ecchymotiques constituent les lésions les plus fréquemment observées selon les données de la littérature [5, 7, 8]. Chez notre patiente, les lésions étaient bulleuses et purpuriques. En effet, dans cette pathologie factice, le caractère bulleux des lésions a été rarement rapporté dans la littérature et peut être induit par une variété de techniques [4, 6]. Cependant, il semble en général difficile d'identifier le mécanisme pathogénique responsable de la formation des lésions [4]. Notre patiente utilise probablement une plante corrosive pour créer ses lésions (données non avouées). Le tableau est souvent stéréotypé avec des lésions bizarres, linéaires ou géométriques aux contours nets, situées sur des zones facilement accessibles (toujours respect du dos). Les lésions peuvent être uniques ou multiples, unilatérales ou bilatérales, symétriques ou non. Le plus souvent, ces lésions siègent au niveau de la face, du thorax, et des extrémités. Chez notre patiente qui est droitière, les lésions sont toujours unilatérales gauches avec respect du dos. Néanmoins, la localisation particulière au niveau des seins peut être très déroutante [4, 8]. Le début est généralement brutal mais flou. De plus, il existe une amélioration ou une guérison des lésions sous occlusion ou plâtre, avec une grande tolérance pour des lésions très affichantes. Ainsi, le diagnostic ne peut se faire que devant un faisceau d'arguments après avoir éliminé les diagnostics différentiels notamment une dermatose connue et les autres diagnostics différentiels psychiatriques [3, 5]. Si la cause sous-jacente n'est pas apparente, le diagnostic d'une pathologie factice doit toujours être envisagé. Chez notre patiente, le début brutal, l'aspect clinique, l'absence de lésions spécifiques à l'examen histologique, la cicatrisation rapide des lésions uniquement sous traitement local occlusif et leur récurrence malgré ces soins adaptés était en faveur d'une pathologie factice. Cependant, le rôle important du secret, bien qu'il y ait une volonté inconsciente de « délivrer un message », ne doit jamais être oublié [3]. Sur le plan nosographique, on pourrait situer la pathomimie dans le domaine des états limites, à la limite entre névrose et psychose [2, 3, 9]. Les traits psychopathologiques qui peuvent conduire à un tel comportement sont souvent difficiles à appréhender. En fait, trois types de personnalité pathologique semblent pouvoir être retrouvés chez les pathomimes: le premier type rassemble des sujets bizarres, excentriques se rapprochant des personnalités paranoïaques et schizophrènes. D'autres pathomimes, instables, capricieuses, et émotives, se rangeraient plutôt parmi les personnalités hystériques, borderlines ou narcissiques. Enfin, certaines pathomimes sont surtout anxieuses à personnalités évitantes, dépendantes, obsessionnelles compulsives. Notre patiente fait partie de la dernière catégorie de personnalité pathologique [3, 5]. Des éléments dépressifs coexistent parfois, comme chez notre patiente.

Il existe une sorte d'association paradoxale entre une demande de soins et induction de lésions, entre une demande de soins mais pas de demande de guérison, une souffrance sincère mais une manipulation du médecin sur les lésions. Quoi qu'il en soit, il n'y a pas de motif rationnel, mais des mécanismes plus ou moins inconscients qui conduisent à afficher sur la peau sa souffrance psychique [3]. Tel était le cas de notre patiente qui s'inflige des lésions après chaque conflit familial. L'attitude à adopter face à de tels patients est difficilement codifiable. Leur prise en charge demande un investissement pluridisciplinaire le plus précoce possible [9]. La collaboration entre dermatologue et psychiatre est essentielle afin d’élaborer un projet thérapeutique commun, et ce en amont de la rencontre du patient avec le psychiatre [6, 9]. Le dermatologue a un rôle très important et son attitude doit être adaptée car elle conditionne l’évolution [3]. La prévention et le traitement des lésions par des pansements et des soins adaptés, en motivant le patient, sont importants. Curieusement, les soins sont faits consciencieusement par les pathomimes, même si elles continuent à créer de nouvelles lésions, comme c’était le cas de notre patiente [3]. La place de l'hospitalisation est à discuter. Il ne doit pas s'agir d'un abandon, mais d'une prise en compte de la souffrance, ou tout au moins c'est le message qu'il faut tenter de faire passer. Le principal intérêt de l'hospitalisation est l’éloignement de l'entourage. Des antidépresseurs peuvent être prescrits s'il existe des symptômes dépressifs, mais ce sont les seuls psychotropes qui puissent être utile, tel était notre cas [3, 9]. En revanche, les psychothérapies d'inspiration analytique ont toute leur place [3, 10]. Dans notre cas, la pathomimie s'associe à des troubles anxieux et dépressifs très importants en relation avec plusieurs stresseurs: situation familiale conflictuelle (avec le frère), deuil pathologique, sentiment d'insécurité, de solitude et de malaise suite au décès du père, angoisse de maladies graves (parents proches décédés de cancer). Le traitement médical (antidépresseurs et anxiolytiques) associé à une prise en charge psychologique de type thérapie cognitivo-comportementale peut aider cette patiente à contrôler son comportement et éviter les récidives qui sont fréquentes dans ce type de pathologie. Cependant, notre patiente a été perdue de vue et n'a pas continué le suivi psychiatrique. En effet, dans notre contexte marocain, la prise en charge thérapeutique reste très difficile en raison de l'habituelle opposition des sujets atteints, et même de leur famille qui refusent ce diagnostic [5].

## Conclusion

La prise en charge des pathomimies est complexe. Il semble important de faire le diagnostic de cette maladie de manière précise car les diagnostics différentiels conduisent à des prises en charge bien différentes. Le pronostic est très mal connu. Le lien entre la pathomimie et un événement précis, l’âge jeune, une personnalité dépendante ou hystérique, des changements positifs dans les relations affectives, la qualité de la coordination des soignants, la qualité de la relation avec un psychothérapeute et le début précoce du traitement peuvent constituer des facteurs positifs. La prise en charge de notre patiente par la thérapie cognitivo-comportementale est une première expérience qui permettra par la gestion des troubles anxieux et dépressifs de contrôler sa pathomimie, malgré l'absence de recul.
